# Primary tooth emergence in young children is associated with a stress biomarker

**DOI:** 10.1017/S2040174426100762

**Published:** 2026-08-12

**Authors:** Dorota Kopycka-Kedzierawski, Changyong Feng, Patricia G. Ragusa, Kim Flint, Gene Watson, Cynthia Wong, Ronald J. Billings, Thomas G. O’Connor

**Affiliations:** 1 Eastman Institute for Oral Health, University of Rochester, USA; 2 Departments of Psychiatry, Neuroscience, Obstetrics and Gynecology, University of Rochester, USA

**Keywords:** Oral health, early childhood caries, stress, cortisol, developmental aging, International Caries Detection and Assessment System (ICDAS)

## Abstract

Early tooth emergence may reflect developmental stress exposures and compose part of a broader set of growth measures that may be accelerated in response to early stress exposure. We test the hypothesis that timing of primary tooth emergence is associated with stress exposure and stress biology in young children. Tooth emergence was tracked prospectively in a diverse sample of initially caries-free 1–3 year-olds; socio-economic and psychosocial determinants of oral health, oral microbiology, and salivary stress markers were assessed at baseline and at 6-monthly intervals for 2 years. The number of tooth surfaces observed at the initial assessment in caries-free children was associated with a stress-related pattern of salivary cortisol reactivity: diminished cortisol response to a laboratory stressor was strongly associated with the number of surfaces (estimate −6.51 [SE 1.90], 95% CI −10.23–−2.79, p < 0.001), after adjusting for covariates. This effect was also found in longitudinal analyses across the two-year study period. The findings provide novel evidence that a marker of stress biology is positively associated with tooth emergence in young children and may reflect additional developmental and biological influences that underlie oral health in children.

## Introduction

Tooth emergence of primary teeth in young children is part of a synchronized physiological process involving osteoclastogenesis in the dental follicle, activity of parathyroid hormone-related protein, and alteration of soft tissue and immediate mandibular mechanics^1–4^; it may be part of a broader maturation pattern of bone growth. Primary tooth emergence follows a highly ordered pattern, but multiple studies indicate considerable variability in the timing – if not the order – of primary teeth before its completion by approximately three years of age.^5,6^ The current study tests the hypothesis that variation in the timing of tooth emergence is predictable from socio-demographic, psychosocial, and stress biology.

Early tooth emergence has particularly relevance to clinical and public health. One reason is that it may predict risk of early childhood caries (ECC)^7,8^ (but see^9,10^), perhaps caused by a longer exposure period for tooth surfaces to attract pathogenic bacteria such as *S. mutans*. Likely because it reflects broader growth and maturational mechanisms, variation in the timing of tooth emergence is under genetic control^11^ but may be delayed by low birth weight, poor nutrition, and prematurity (although perhaps not after age correction)^12,13^ and, inversely, accelerated by early weight gain.^14,15^ More novel is an emerging developmental aging hypothesis from the developmental origins field: social and/or economic deprivation may predict accelerated dental development. The hypothesis that early adversity is associated with *advanced* dentition follows on from research evidence suggesting that early stress exposure is associated with *accelerated* aging according to a number of diverse developmental aging markers, including age at menarche, telomere length, and epigenetics, e.g.^16^ Leverage for testing a developmental aging hypothesis in the current study derives from several research design strengths, including a prospective 2-year study period of initially caries-free 1–3 year-olds; collection of oral microbiology; detailed measures of socio-economic adversity and leading parent-reported measures of psychosocial stress; and a leading biological marker of stress biology in young children, salivary cortisol reactivity, collected in response to a laboratory stressor.

## Material and methods; sample

A cohort of confirmed caries-free pre-school children was recruited from a university-based community pediatric dental clinic in the Northeast US; the longitudinal study was conducted between February 2016 and February 2021. Inclusion criteria were (a) child age of 1–3 years at enrollment, (b) Medicaid and Child Health Plus eligible, (c) primary caregiver age of 18 years or older, (d) sufficient understanding of English to complete study procedures and measures. Exclusion criteria were (a) major medical problems and (b) evidence of dental caries at enrollment. Children on antibiotics were not excluded but a study visit was (re-)scheduled to occur at least 30 d from end of the antibiotic exposure (i.e., in order for valid collection of microbiology data). The study was approved by the local Institutional Review Board and written informed consent was obtained from a primary caregiver; study participants were reimbursed.

## Procedure and measures

Children and a primary caregiver were seen at a university-based pediatric community clinic at enrollment and then at 6-month intervals for 2 years (i.e., up to 5 visits in total). Each visit consisted of behavioral assessments, parent-completed questionnaires, saliva collection, and a formal dental examination; each visit lasted approximately 2 h.

### Oral examination

At each visit children were examined by a calibrated pediatric dentist using standard protocol and procedures^17^; children were confirmed clinically caries-free at baseline according to the International Caries Detection and Assessment System (ICDAS) (ICDAS = 0, *d* = 0, *f* = 0, *m* = 0). At each follow-up assessment, we recorded the presence/absence of ECC and ICDAS severity on the tooth surface level recorded as the number of non-cavitated (ICDAS = 1 and ICDAS = 2) and cavitated (ICDAS ≥ 3), filled (due to caries) and missing tooth surfaces (due to caries). We also recorded surfaces that were missing for reasons other than dental caries (exfoliation or trauma) and unerupted surfaces. The total number of surfaces was used as a measure of tooth emergence because it is more sensitive than the number of teeth.

### Child stress physiology

Salivary samples were collected prior to and 15’ and 30’ following a standard laboratory stressor; a parent-child separation stress was used at yearly visits 1, 3, 5^18–20^; a parent-child interaction was used at visits 2 and 4. Samples were collected using the Salimetrics Children’s Swab (SCS) which are designed for the collection of saliva from children under the age of six years. The SCS is long enough to allow one end of the swab to be held while it is in the child’s mouth, eliminating any choking hazard. Following saliva collection, samples were transported to the laboratory on ice and stored at −20C until processed in triplicate per manufacturer’s instructions using the Expanded Range High Sensitivity Salivary Cortisol Enzyme Kit (#1–3002) (Salimetrics). Standard curves were fit to a 5 Parameter Logistic for determination of cortisol concentration in saliva (GainData Elisa Calculator, Arigo Biolaboratories). The inter-and intra-assay coefficients of variability for cortisol determinations were 10.7% and 1.8%, respectively. We followed established practice to avoid confounds and contamination (e.g., saliva collected for the laboratory stressor was administered at the beginning of the visit; no food or beverages were consumed within 30 min of saliva collection). We considered both area under the curve with respect to increase (AUCi) as the primary measure and consider AUCi with and without adjusting for pre-stressor (given the universal observation of an modest inverse association between pre-stressor concentration and change associated with the stressor); area under the curve with respect to ground (AUCg) was also considered. Time of first sample collection was included as a covariate in cortisol analyses. Cortisol is reported as the concentration (mcg/dL) in saliva.

#### Oral microbiology

was assessed from a sample of approximately 2 mL of saliva collected prior to the dental examination and separate from the stressor-related salivary cortisol collection at each visit. Microbiological profiles included *mutans streptococci*, *Lactobacilli* (LB), *Candida* species using established microbiological procedures.^21^ Whole, stimulated, saliva samples were obtained through a disposable saliva ejector attached to a 50 ml sterile centrifuge tube, which in turn was attached to a vacuum pump.^22^ All salivary samples were processed the same day using an Autoplate Spiral Plating System (Advanced Instruments, Inc.). Saliva samples were evaluated for MS, LB, and Candida species using Mitis Salivarius agar plus bacitracin, Rogosa, and CHROMagar respectively. For dispersion of cell clumps and dechaining of streptococci, a 2 ml aliquot of saliva was sonicated (three 10 s sonic bursts, 100 W of peak power). The suspension was serially diluted (10-fold dilutions) in phosphate buffer and 50 μl aliquots uniformly plated on (i) Mitis Salivarius agar (Becton-Dickinson) supplemented with 20% sucrose and bacitracin (0.2 U/ml), to determine the presence of MS, (ii) Rogosa agar (Oxoid) to evaluate the presence of *Lactobacilli*, (iii) CHROMagar (BBL) supplemented with 0.1 mg/ml chloramphenicol to determine the presence of Candida spp., and (iv) tryptic soy agar supplemented with 5% sheep blood to enumerate the total microflora. MS plates were incubated at 37C in a 5% CO2 atmosphere, Rogosa plates were incubated anaerobically at 37C, CHROMagar plates were incubated aerobically at 37C and duplicated blood agar plates incubated at 37C both under aerobic and anaerobic conditions. All dilutions were plated in triplicates and the plates incubated under the described conditions for 72 h before colonies were counted. The number of *mutans streptococci*, *Lactobacilli*, *Candida* and total microflora were expressed as colony-forming units (CFUs) per ml of saliva. Detailed procedures for collection, storage, and analysis of oral microflora are well-established and described elsewhere.^22^ Salivary samples were processed between 1 and 4 h after collection, as there is no significant loss of numbers of total viable flora during the first 24 h.^22^ Measures of oral microbiology were log-transformed prior to analyses.

#### Child and family stress exposures

were based on parent-reported measures at each visit from six widely used inventories assessing different sources of stress exposures: caregiver depression based on the Center for Epidemiologic Studies Depression Scale^23^; anxiety and worry based on the Penn State Worry Questionnaire^24^; alcohol use from the Alcohol Use Disorders Identification Test^25^; stressful life events from a list of standard high-stress conditions (e.g., losses of income, health problems^26^); household disorganization and confusion from the Confusion, Hubbub, and Order Scale^27^; violence exposure based on the psychological aggression and physical assault subscales of the Conflict Tactics Scale^28^ (following guidelines, this scale was administered yearly rather than at 6-monthly intervals). Following decades of clinical research practice in psychosocial studies^29^ and current practice in clinical health studies, e.g.,^30,31^ a composite measure from standardized scores of each of the six scales was created at each visit.

### Socio-demographic variables and covariates

Socio-demographic variables included the child’s and parents/primary caregivers’ age, race, ethnicity, and sex; insurance status; parent/primary caregiver educational attainment and occupation; and income. Favorite snacks/drinks and oral health behaviors were identified via parental questionnaire from a validated measure assessing child’s eating and drinking habits, snacking choices, sippy cup use, and the type and the amount of beverages and snacks consumed by the children; we also assessed current breast feeding and formula feeding practices and oral hygiene regimen.^32^ Also included was parent-reported history of prematurity (<37 weeks) and current smoking status (scored as yes versus no).

## Data analysis

Descriptive data and attrition analysis across the two-year assessment period are presented first. Subsequent analyses employ bivariate and multivariable statistics to examine associations between the number of tooth surfaces present and socio-demographic, psychosocial, and biological measures. Prediction analyses were based on generalized estimating equations (GEE), which employs a regression model framework that accommodates repeated measurement within subject. Several socio-demographic covariates were considered on an *a priori* basis following previous research: prematurity, parental smoking, child sex, age, race/ethnicity, education, insurance status; other factors were also considered as covariates if there was reliable evidence of their association with exposure or outcome variables.

## Results

### Descriptive and preliminary analyses

A total of 189 confirmed caries-free pre-school children were enrolled. Sample socio-demographic characteristics are displayed in Table 1, which indicate that the participating families are ethnically and racially diverse and at relatively high socio-demographic risk, e.g., based on parental educational attainment and Medicaid eligibility status. Of the *n* = 189 participants at baseline, retention was 72% from initial visit to 1 year and 95% from year 1 to year 2. There was no reliable evidence that demographic, oral hygiene, or psychosocial factors in Table 1 predicted retention through the 2-year study period.

At the initial assessment, the number of tooth surfaces ranged from 16 to 88, and was strongly associated with child age (Means [SD]: <2 years = 57, [17] *n* = 57; 2–3 years = 80, [10] *n* = 81; >3 years = 87, [3] *n* = 51; *F*(2, 186) = 105.6, *p* < 0.0001) but not child sex (Mean [SD] males = 76, [17] *n* = 98; females = 75, [17] *n* = 91; *F*(1, 187) = 0.08, *p* = .78) with no evidence of a child age X sex interaction (estimate for the interaction = 0.04 [SE 0.19], *p* = 0.73) (see Appendix 1 for the number of surfaces at each study assessment by child age). As expected, the number of surfaces detected over the 2-year study period increased but strongly depended on child age at baseline (see Fig 1). GEE results confirmed that child age (estimate 1.43 [SE 0.13], 95% CI 1.17–1.68, *p* < 0.001) and time or visit (estimate 13.19 [SE 1.13], 95% CI 1.13–10.97, *p* < 0.001) were associated with the number of tooth surfaces observed across the study period, but that these effects were qualified by a significant child age and visit interaction (estimate −0.34 [SE 0.03], 95% CI−0.41–−0.28, *p* < 0.001). Consistent with this observation is the finding that the intraclass correlation coefficient (ICC) for the number of surfaces, as an index of similarity in the number of surfaces of over time, was modest overall (ICC 0.25) and nil for children who were <2 years at enrollment (ICC 0.02). Appendix II, which reports the small number of children at each visit with a lost tooth (for any reason), indicates that tooth loss is unlikely to confound these results (and so tooth loss was not considered further). Preliminary analyses also indicated that number surfaces at baseline were unrelated to prematurity, likely a consequence of the small number of affected cases, prenatal smoking, history of breastfeeding, or nutrition/dietary habits.

### Does the number of surfaces at baseline associate with socio-economic, psychosocial, oral microbiology, and stress biology?

Bivariate associations between the number of surfaces at baseline and socio-demographic, psychosocial, and biological measures are provided in Table 2. Three reliable effects emerged. Greater number of surfaces at baseline or initial visit – when all children were caries free – was significantly positively associated with the presence of *S. mutans* (0.55 [SE 0.27], 95% CI 0.02–1.08, *p* < 0.05) and inversely associated with *Lactobacilli* (−1.51 [SE 0.76], 95% CI −0.30–−0.03, *p* < 0.05). In addition, decreased cortisol response to the stressor, indexed by (a negative or less positive) AUCi, was strongly associated with the number of surfaces (estimate −6.51 [SE 1.90], 95% CI −10.23–−2.79, *p* < 0.001). In contrast, neither socio-demographic nor parent-reported psychosocial stress was reliably associated with the number of surfaces at baseline. A multivariable analysis was carried out with a reduced set of covariates (Table 3). Results indicated that AUCi remained a reliable predictor of number of surfaces at baseline. In addition, the presence of *S. mutans* was positively and the presence of *Lactobacilli* was inversely associated with the number of tooth surfaces at the initial (baseline) assessment.

### Longitudinal prediction analyses of tooth surfaces across the 2-year study period

The above analyses at baseline were then extended longitudinally to examine if the patterns were robust across the 2-year period of follow-up. Bivariate associations between the number of tooth surfaces across the 2-year study period and socio-demographic, psychosocial, oral biology, and stress biology measures are provided in Appendix III. Results from the longitudinal data were nearly identical to results from the initial number of tooth surfaces at baseline (Table 2), with one notable exception: the psychosocial stress composite was strongly positively associated with the number of tooth surfaces when data across the two-year period were analyzed. Results from a multivariable extension of the longitudinal patterns are provided in Table 4, which demonstrates that cortisol reactivity continued to be a reliable predictor of the number of tooth surfaces using data collected across the 2-year study period. In addition, older age at enrollment (measured in years) and visit number were reliably predictors of tooth surfaces.

### Prediction from ECC risk from the number of surfaces

Over the 2-year follow-up, approximately 40% of the children converted to caries-active status. Longitudinal analyses indicated that the number of surfaces at baseline was non-significantly positively associated with ECC risk (OR = 1.003, 95% CI: 0.980–1.027, *p* = 0.790) after adjusting for child age, with no evidence of age moderation (OR = 0.998, 95% CI: 0.995–1.001, *p* = 0.217).

## Discussion

Analyses of tooth emergence data on an initially caries-free sample of 1–3 year-olds indicated that a leading measure of stress biology, diminished cortisol reactivity to a laboratory stressor, was associated with advanced dental development, indexed by number of surfaces as determined from a formal pediatric oral examination. This association remained in longitudinal analyses that incorporated two years of data. Additionally, the number of surfaces was associated with the presence of *S. mutans* in both cross-sectional and longitudinal analyses, which further supports the hypothesis that (comparatively) early tooth emergence may be a caries risk because of the longer period of attraction to cariogenic agents. These results add significantly to research on the developmental origins and clinical implications of the timing of tooth emergence in young children and the impact on health more broadly.

### Tooth emergence as a developmental aging variable

Timing of tooth emergence varies widely; for example, one study reported that the average age at emergence of the right central maxillary incisor varied in children from different countries from 8.19 to 11.40 months.^6^ One way that timing of tooth emergence – and most especially early tooth emergence – presents clinical and public health concerns is as a predictor of ECC, one of the most common chronic diseases affecting children^33^ that has lasting influence on health, social, and academic outcomes.^34^ Our findings add to this research in several ways. Most fundamentally, in analyses of baseline data in *caries-free children*, we found that the number of tooth surfaces was associated with *S. mutans*. That observation supports the contention that the number of surfaces does attract *S. mutans*, likely because they attach to tooth surfaces due to the presence of a salivary pellicle that provides specific binding sites for its adhesins.^35^ This association remained in longitudinal analyses, indicating that, over time, those children with more tooth surfaces reliably had greater presence of cariogenic bacteria. An inverse association with *Lactobacilli*, which is generally regarded as a marker of disease progression, may not have a ready interpretation in caries-free children at the initial assessment. Furthermore, a lack of association in longitudinal analyses suggests that the association at baseline may not be a reliable finding.

The number of surfaces was reliably associated with *S. mutans* and may constitute a direct effect. In contrast, the association between number surfaces and ECC likely reflects a less direct process, which reinforces the view that associations between early tooth emergence and ECC may be complex – as implied by inconsistent findings reported in the literature. In the current study we failed to find that tooth surfaces predicted ECC onset, and that was also the case when the sample was stratified by child age. Warren et al.^10^ suggested that this association may be age-dependent and most evident in longitudinal studies, which could explain some of the discrepancies across studies.

Forensic medical and anthropological evidence makes clear that tooth emergence is a reliable marker of chronological age. Nonetheless, there is variation in age of tooth emergence, suggesting that tooth emergence could reflect accelerated (more tooth surfaces observed than expected according to chronological age) or decelerated (fewer tooth surfaces than expected based on chronological age). Set against the null hypothesis that this variation in tooth surfaces *for a given age* is random is a novel alternative developmental origins hypothesis that early tooth emergence, as an index of accelerated dental growth, is associated with early stress exposure. Available evidence to date has been indirect or limited. For example, cross-cultural variation in tooth emergence^6,36^ may imply social environmental influence but does not offer a direct test. More direct support for a stress-induced accelerated dental development hypothesis was provided by Ntani et al.,^37^ who reported that an index of multiple deprivation was associated with earlier tooth emergence at two years of age. Furthermore, two studies of older children found that reports of early life stress associated with earlier emergence of molars from middle childhood.^38,39^

Our findings importantly extend these reports by demonstrating that a leading biomarker of early and chronic stress exposure, dampened cortisol response to a laboratory stressor,^19, 40, 41^ predicted a higher the number of primary tooth surfaces in young children. The prediction at the initial assessment remained after adjusting for important covariates, including child age and *S. mutans*; furthermore, the association at baseline is also not confounded by caries status because all children were confirmed caries-free at the baseline assessment. Moreover, the effect was evident in longitudinal analyses that assessed tooth surfaces over the two-year study period. The finding provides perhaps the strongest evidence to date that accelerated dental development may be associated with early stress biology – and that this effect is evident early in development.

One of the more notable findings in the developmental origins of health and disease literature concerns rate of development; in almost all published examples, stress exposure was associated with accelerated growth. Several lines of research suggest that markers of development in pediatric populations – as varied as microbiome composition, DNA methylation patterns, age at menarche, brain imaging patterns, and T cell populations – may reflect individual differences in developmental aging and be accelerated in response to early stress.^42,43^ Biological mechanisms underlying accelerated aging are unclear, although evolutionary explanations have been offered, e.g., more rapid physical development may confer competitive advantage or lead to increased physical maturation and early reproductive success. Notably, stress biology, including cortisol, the focus of the current paper, figures prominently in hypotheses of stress exposure and accelerated aging – including in studies of rapid early growth.^44^ A dampened stress response, which predicted increased number of tooth surfaces in the current study, is believed to reflect early and/or chronic stress, perhaps reflecting down-regulation of receptor sensitivity following early and prolonged stress exposure. However, the prediction from child cortisol may also reflect other biological processes, as glucocorticoids have many biological functions, and these could be involved with early tooth emergence. Replicating the current results, including other markers of stress biology, linking tooth emergence with other biological aging markers, and extending longitudinal analyses to permanent teeth are next directions for this line of investigation.

Alongside the prediction from stress biology, there was modest evidence that a composite of parent-reported psychosocial stress was associated with increased number of tooth surfaces – also suggesting that stress may accelerate tooth emergence. That the prediction was not evident at baseline and did not survive in multivariable analyses may simply indicate a weak effect; this association was in any event less robust than the measure of stress biology, which may be more proximal to the biological mechanisms associated with dental and bone growth. Consistent with prior reports,^6,37^ we did not find that socio-demographic-linked stress measures associated with the number of surfaces. That notable non-finding may underscore the value of directly measuring stress exposure and stress biology rather than inferring stress exposure from socio-demographic status. Alternatively, a lack of association with socio-economic status may reflect the high proportion of low-resource families that we sampled.

The current study has several limitations. For example, the number of teeth or, as in this study, the number of tooth surfaces, may be a less accurate index of physiological maturity than dental calcification. Replication of findings using other methods for assessing dental aging, such as X-ray for calcification or the Demirjian method,^45,46^ is needed. These alternative methods may have advantages for assessing dental aging, although they may have significant limitations, such as research ethics concerns for using X-ray in infants and young children for research purposes only; and the application of the Demirjian method may not be suitable for very young children. In addition, the measure of tooth emergence was limited to primary teeth. The current study did not have access to some factors previously associated with tooth development, such as prenatal nutrition and early growth, which could be confounds. Also, the study over-sampled low-resource families, which limited our ability to assess tooth emergence across a broad array of socio-economic conditions. Set against these limitations are several strengths of the study, including a formal pediatric dental examination using the ICDAS system; detailed collection of oral microbiology, psychosocial risk exposure and measures of stress biology; consideration of tooth loss in analyses of the number of tooth surfaces; and a prospective longitudinal design that sampled initially caries-free children.

The current report is part of an effort to incorporate oral health into broader pediatric and preventive health and development field,^47,48^ and to examine how common developmental exposures may underlie overlapping oral, behavioral, and physical health outcomes in children. Our finding that a leading biomarker of child behavioral health predicted a significant measure of dental development and perhaps oral health is a novel but unlikely unique example. The current results also identify specific clinical health implications of the timing of tooth emergence: early onset of dentition may require additional oral health intervention to offset greater exposure to oral microbiological risk for ECC. Finally, studies are needed that incorporate growing lessons about accelerated dental development and biomarkers of risk into prevention interventions for oral health that target behavioral approaches^49^ and commence in pregnancy.^50^

## Supplementary Material

Supplementary material

**Supplementary material.** The supplementary material for this article can be found at https://doi.org/10.1017/S2040174426100762.

## Figures and Tables

**Figure 1.**
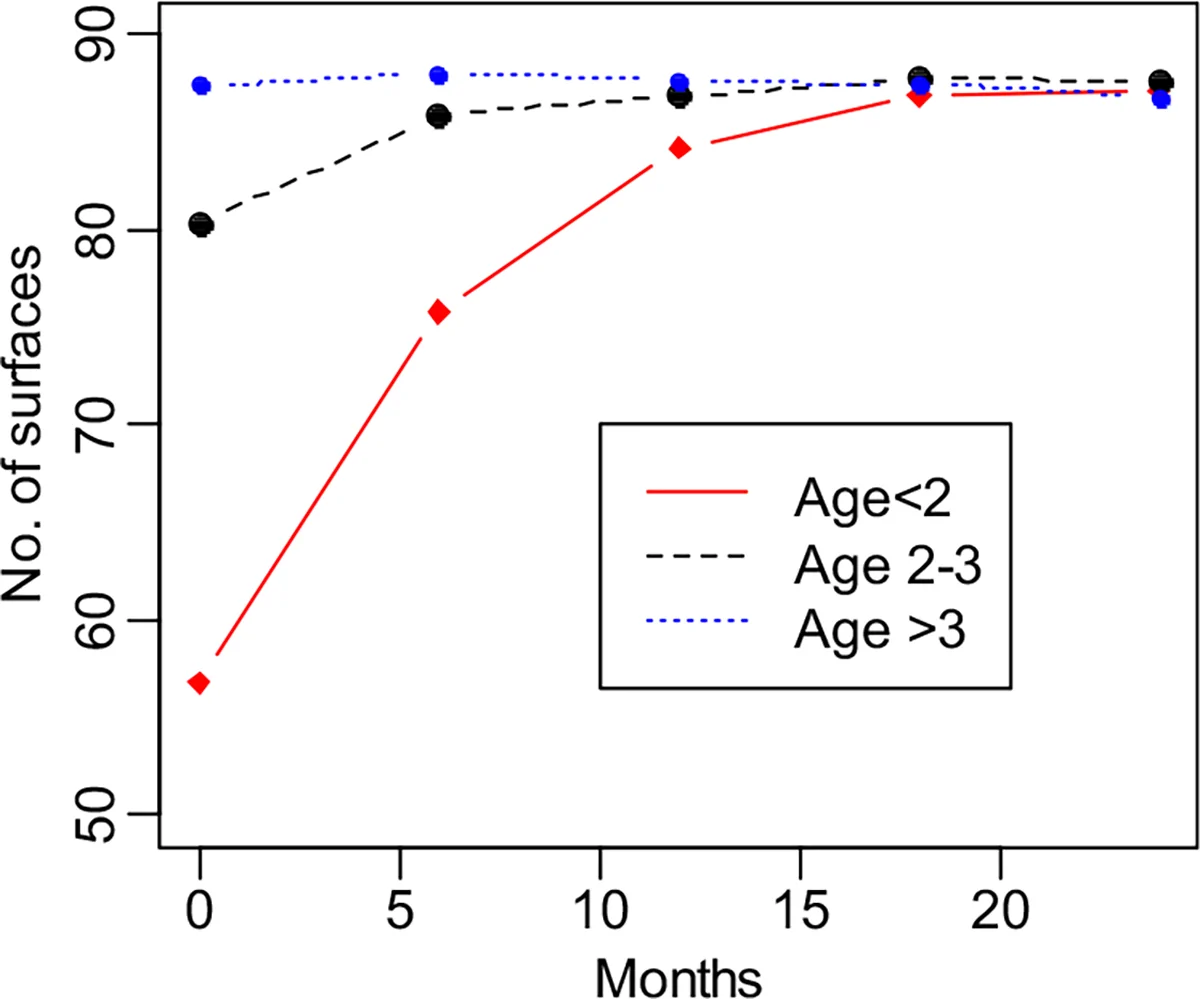
Number of tooth surfaces across the 24-month follow-up period according to child age at enrollment.

**Table 1.** Social and demographic characteristics of the sample at enrollment

	Mean (SD)	*n* (%)
Maternal education		
< High school		17 (9.2)
High school		76 (41.3)
GED		29 (15.8)
Some college		47 (25.5)
Univ degree or more		15 (8.2)
Marital/cohabiting status		
Single		104 (55)
Married/cohabiting		85 (45)
Employed		108 (57.1)
Smoking in the household (yes)		38 (20)
Number of people/house	4.1 (1.5)	
Child sex (female)		91 (48.2)
Child age (months)	29.5 (9.1)	
Child race		
Black/African-American		76 (40.2)
White		35 (18.5)
Mixed/other		78 (41.3)
Child ethnicity (Hispanic)		50 (26.5)
Premature birth		31 (16.4)
Child dental insurance		
Medicaid		143 (75.7)
Child health plus		35 (18.5)
Private		10 (5.3)
None		1 (.5)

**Table 2.** Bivariate associations with number of surfaces at baseline

Parameter	Estimate	Standard error	95% confidence interval		*p*-Value
Child age at enrollment (years)	1.3475	0.1005	1.1506	1.5445	<0.0001
Child sex	−1.8739	2.7244	−7.2137	3.4658	0.4926
Education					
Educ 2 vs 1	−3.7706	4.9612	−13.4945	5.9534	0.4484
Educ 3 vs 1	−1.6553	5.7154	−12.8576	9.5470	0.7725
Educ 4 vs 1	−8.2216	5.2531	−18.5178	2.0745	0.1197
Educ 5 vs 1	−6.2473	6.4942	−18.9758	6.4813	0.3376
Dental insurance	−1.3094	2.3253	−5.8670	3.2481	0.5742
*S mutans* (lg)	0.5504	0.2700	0.0211	1.0797	0.0433
*Lactobacillus* (lg)	−1.5094	0.7572	−2.9935	−0.0254	0.0481
*Candida* (lg)	−0.1547	0.5365	−1.2062	0.8968	0.7735
Psychosocial stress composite	2.4509	2.7208	−2.8819	7.7837	0.3691
AUCi	−6.5128	1.9009	−10.2385	−2.7871	0.0008

*Note:* for Educ 1= less than high school graduation (12 years), 2 = high school graduation, 3 = GED equivalency, 4 = some university, 5 = completed university degree or greater; AUCi = area under the curve with respect to increase (see text).

**Table 3.** Multivariable model prediction of number of tooth surfaces at initial assessment

Parameter	Estimate	Standard error	95% CI		Pr > |t|
Child age (enrollment)	1.213	0.099	1.018	1.407	<0.0001
*S mutans* (lg)	0.506	0.181	0.152	0.860	0.006
*Lactobacillus* (lg)	−1.015	0.437	−1.871	−0.159	0.021
*Candida* (lg)	0.130	0.353	−0.561	0.822	0.712
AUCi	−3.046	1.329	−5.650	−0.441	0.023

Note: AUCi = area under the curve with respect to increase; analyses adjust for time of saliva collection (see text).

**Table 4.** Longitudinal multivariable model prediction of number of tooth surfaces

Parameter	Estimate	Standard error	95% confidence interval		*p*-Value
Child age (enrollment)	0.488	0.055	0.381	0.595	<0.0001
*S mutans* (lg)	0.217	0.112	−0.002	0.435	0.052
Psychosocial stress composite	1.345	0.873	−0.366	3.056	0.124
AUCi	−1.968	0.637	−3.216	−0.719	0.002
Visit	2.402	0.350	1.716	3.088	<0.0001

Note: AUCi = area under the curve with respect to increase (see text); analyses adjust for time of saliva collection (see text).
