# Supplementary material for: Primary tooth emergence in young children is associated with a stress biomarker
Source: J Dev Orig Health Dis. Author manuscript; Available in PMC 2026 Oct 1. (PMC13628187; doi:10.1017/S2040174426100762)
Supplement: Supplementary file 1 [file NIHMS2210085-supplement-Supplementary_material.docx]

Appendix 1. Number of surfaces at each visit, stratified by age at study entry.

| **Age group** | **Visit** | **N** | **Mean** | **Std Dev** | **Minimum** | **Maximum** |
| --- | --- | --- | --- | --- | --- | --- |
| <2 years | **1** | 57 | 56.8 | 17.5 | 16 | 88 |
|  | **2** | 50 | 75.8 | 11.8 | 44 | 88 |
|  | **3** | 45 | 84.2 | 6.1 | 68 | 88 |
|  | **4** | 42 | 86.9 | 4.3 | 68 | 88 |
|  | **5** | 33 | 87.2 | 3.4 | 72 | 88 |
| 2-3 years | **1** | 81 | 80.4 | 10 | 42 | 88 |
|  | **2** | 68 | 85.9 | 6 | 64 | 88 |
|  | **3** | 60 | 86.9 | 5.5 | 48 | 88 |
|  | **4** | 51 | 87.8 | 1.1 | 80 | 88 |
|  | **5** | 37 | 87.7 | 1.5 | 80 | 88 |
| >3 years | **1** | 51 | 87.4 | 3.1 | 68 | 88 |
|  | **2** | 45 | 88 | 0 | 88 | 88 |
|  | **3** | 42 | 87.7 | 1.4 | 80 | 88 |
|  | **4** | 38 | 87.5 | 1.7 | 80 | 88 |
|  | **5** | 30 | 86.8 | 3.3 | 76 | 88 |

Appendix II. Number of participants at each visit with any missing teeth due to exfoliation or injury/trauma

| Visit | n |
| --- | --- |
| 1 | 1 |
| 2 | 3 |
| 3 | 5 |
| 4 | 5 |
| 5 | 7 |

Appendix III. Longitudinal Bivariate Associations with Number of Surfaces

| Parameter | Estimate | Standard error | 95% Confidence Interval | | P-value |
| --- | --- | --- | --- | --- | --- |
| Child age | 0.468 | 0.049 | 0.373 | 0.564 | <.0001 |
| Child sex | -0.043 | 1.064 | -2.129 | 2.043 | 0.968 |
| Education |  |  |  |  |  |
| Educ 2 vs 1 | -1.305 | 1.177 | -3.611 | 1.001 | 0.267 |
| Educ 3 vs 1 | -1.071 | 1.591 | -4.189 | 2.046 | 0.501 |
| Educ 4 vs 1 | -2.697 | 1.533 | -5.702 | 0.308 | 0.079 |
| Educ 5 vs 1 | -2.554 | 1.949 | -6.375 | 1.266 | 0.190 |
| Dental insurance | -0.066 | 0.789 | -1.612 | 1.480 | 0.933 |
| *S mutans* (lg) | 0.348 | 0.145 | 0.064 | 0.632 | 0.017 |
| *Lactobacillus* (lg) | 0.091 | 0.210 | -0.320 | 0.502 | 0.665 |
| *Candida* (lg) | -0.205 | 0.205 | -0.607 | 0.198 | 0.319 |
| Psychosocial stress composite | 2.669 | 0.723 | 1.253 | 4.086 | <0.001 |
| AUCi | -3.131 | 0.729 | -4.560 | -1.701 | <.0001 |

Note: for Educ 1= less than high school graduation (12 years), 2 = high school graduation, 3 = GED equivalency, 4 = some university, 5 = completed university degree or greater; AUCi = area under the curve with respect to increase (see text).
